# What is the need and why is it time for innovative models for understanding lung repair and regeneration?

**DOI:** 10.3389/fphar.2023.1130074

**Published:** 2023-02-13

**Authors:** Daniel J. Weiss

**Affiliations:** University of Vermont, Burlington, VT, United States

**Keywords:** lung, cell therapy, bioengineering, lung disease, stem cells

## Abstract

Advances in tissue engineering continue at a rapid pace and have provided novel methodologies and insights into normal cell and tissue homeostasis, disease pathogenesis, and new potential therapeutic strategies. The evolution of new techniques has particularly invigorated the field and span a range from novel organ and organoid technologies to increasingly sophisticated imaging modalities. This is particularly relevant for the field of lung biology and diseases as many lung diseases, including chronic obstructive pulmonary disease (COPD) and idiopathic fibrosis (IPF), among others, remain incurable with significant morbidity and mortality. Advances in lung regenerative medicine and engineering also offer new potential avenues for critical illnesses such as the acute respiratory distress syndrome (ARDS) which also continue to have significant morbidity and mortality. In this review, an overview of lung regenerative medicine with focus on current status of both structural and functional repair will be presented. This will serve as a platform for surveying innovative models and techniques for study, highlighting the need and timeliness for these approaches.

## Overview of lung regenerative medicine

As lung diseases and critical illnesses have diverse pathophysiologies and manifestations, multiple potential regenerative medicine-based therapeutic approaches are being investigated. For functional repair, ARDS and sepsis/septic shock offer paradigms for disease entities marked by acute high intensity inflammation and associated injuries. A range of pharmacologic agents including monoclonal antibodies, corticosteroids, non-steroidal anti-inflammatory agents have only modestly improved morbidity and mortality if at all and current strategies are primarily focused on supportive care (Matthay et al., 2019a). Acknowledging that some ARDS survivors develop long term fibrotic changes, focus for new therapeutics is on novel approaches that can counter inflammation. This includes consideration for patients with respiratory failure and ARDS in the setting of SARS CoV2 infection.

### Cell-based therapies: Functional repair

One new promising approach centers on cell-based therapies, predominantly utilizing mesenchymal stromal cells (MSCs) (Walter et al., 2014). MSCs are multipotent progenitor cells with the potential to secrete a spectrum of biologic mediators, such as anti-inflammatory cytokines, angiogenic factors, anti-bacterial peptides, and extracellular vesicles (EVs) (reviewed in 3). Originally isolated from bone marrow, MSCs can be isolated from a wide range of tissues including adipose, placental, cord blood, and from the lung itself (Walter et al., 2014). The general approach is to isolate MSCs from the source tissue and expand in culture for subsequent investigation and potential therapeutic effects. Following systemic administration, MSCs primarily lodge in the pulmonary capillary system, through as yet poorly understood interactions with the endothelium. They do not engraft and are cleared by a range of host immune mechanisms over 1–2 days, although available data suggests that they may persist for up to 3–4 days in the setting of lung inflammation (Allers et al., 2004; Armitage et al., 2018). MSCs express a range of danger and molecular pathogen cell surface receptors, including the Toll-Like receptors and a growing body of literature demonstrates that while in the pulmonary capillary bed, the MSCs respond to specific inflammatory environments by releasing different portfolios of mediators (Romieu-Mourez et al., 2009; Waterman et al., 2010; Kusuma et al., 2017; Abreu et al., 2019; Islam et al., 2019; Enes et al., 2021; Galipeau et al., 2021). The MSCs also constitutively express low levels of cell surface antigens such as human leukocyte antigens (HLA) DR and co-stimulatory molecules such as CD40 (Galipeau and Sensébé, 2018). This has allowed a range of investigations in which allogeneic MSCs have been administered by systemic (intravenous) administration in a wide range of disease models and clinical investigations. However, HLA and co-stimulatory molecules can be upregulated in response to inflammatory signals, notably interferon gamma (IFNγ) (Krampera et al., 2006; Polchert et al., 2008). Accordingly increasing evidence suggests that host responses to the allogeneic MSCs, including MSCs undergoing apoptotic or necrotic changes following administration, may be driving potential therapeutic effects (Galleu et al., 2017; Weiss et al., 2019).

There have been a large number of investigations of both systemic and direct airway administration of MSCs in a wide range of pre-clinical models of lung diseases and critical illnesses. These have been predominantly in rodents but also include large animal models such as sheep and also in explanted human lungs (Ikonomou et al., 2022; Ting et al., 2022). In virtually every case, MSC administration has ameliorated acute, and in some cases chronic, inflammation and injury in models of acute endotoxin and bacterial lung injury, allergic airways inflammation/asthma, bronchopulmonary dysplasia (BPD), COPD, pulmonary hypertension, and sepsis/septic shock (Ikonomou et al., 2022; Ting et al., 2022). The data is less clear for chronic diseases such as pulmonary fibrosis, in part as the available pre-clinical models, for example bleomycin administration in rodents, do not fully replicate human disease. MSC administration during the acute inflammatory phase resulting shortly after bleomycin administration is decreased with subsequent decrease in resulting fibrosis, however, it is less clear as to whether MSCs have benefit in models of established fibrosis. The mechanisms by which the MSCs act in each of these models are not yet fully elucidated and available evidence suggests different mechanisms in the different disease models, consonant with growing appreciation that MSCs will behave differently in varying injuries. Pre-conditioning MSCs, for example by exposure to hypoxia or to different mixtures of cytokines can alter subsequent MSC actions consonant with the observations that MSC behaviors are dictated by the inflammatory environment they encounter (Romieu-Mourez et al., 2009; Waterman et al., 2010; Kusuma et al., 2017; Abreu et al., 2019; Islam et al., 2019; Enes et al., 2021; Galipeau et al., 2021). Further, a growing experience demonstrates that EVs derived from MSCs can mimic many of the beneficial effects of the MSCs themselves (Zhu et al., 2014; Cruz et al., 2015a; Mahida et al., 2020). The mechanisms by which the EVs act are still being elucidated and postulated mechanisms include actions of miRNAs, proteins such as anti-inflammatory cytokines, mitochondria, and lipids contained in the EVs (Abreu et al., 2021).

Nonetheless, these pre-clinical data have provided a platform for a growing number of clinical investigations of MSCs and also MSC-derived EVs in a range of pulmonary diseases and critical illnesses including but not limited to ARDS, asthma, BPD, COPD, IPF, and others [reviewed in (Matthay et al., 2019b; Ting et al., 2022)]. This includes a large number of clinical investigations of MSCs in patients with SARS-CoV2 (COVID-19)-relatedd ARDS in the setting of the COVID-19 pandemic (Dilogo et al., 2021; Lanzoni et al., 2021; Monsel et al., 2022). The majority of these investigations for which published data is available have been phase 1 safety trials and have uniformly demonstrated no acute infusional toxicities and no attributable subsequent serious adverse events. A smaller but growing number of phase 2 efficacy trials have demonstrated efficacy in some cases but not in others. The most extensive experience to date is in investigations of systemic MSC administration in patients with either non-COVID or COVID19-related ARDS with recent meta-analyses of COVID-19 related-ARDS demonstrating significant benefits of MSC administration (Khoury et al., 2020; Qu et al., 2020; Zhu et al., 2021; Kirkham et al., 2022; Lu et al., 2022). Intensive deliberation about the varying outcomes in these trials has highlighted ongoing issues with investigations of MSC administration not just in patients with respiratory diseases or critical illnesses but in a wider range of conditions. These include lack of concordance or clear guidelines as to which source of MSCs may be most effective in any given indication, dose and dosing strategy, and whether freshly thawed (from frozen stocks) or continuously cultured MSCs may be more effective (Francois et al., 2012; Cruz et al., 2015b). These considerations also include growing appreciation that the patient phenotype is critically important in identifying patients more likely to respond to MSCs administration (Matthay et al., 2020). This is in the setting of growing appreciation of different inflammatory phenotypes for patients with ARDS, COPD, and other conditions. For example, a recent trial of systemic administration of bone marrow-derived MSCs in patients with COVID19-related ARDS and respiratory failure requiring mechanical ventilation demonstrated no benefit in survival or other ICU outcome measures across the entire study population (Bowdish et al., 2022). However, a pre-specified subgroup analysis demonstrated significant and substantial improvement in mortality in patients under age 65 (Ting et al., 2022). In a parallel example, systemic administration of allogeneic bone marrow-derived MSCs in COPD patients did result in improvements on lung function (Weiss et al., 2013) but a post-hoc subgroup analysis, in which patients were stratified by entry levels of a circulating inflammatory mediator, C-reactive protein, associated with worse overall clinical outcomes, demonstrated statistically significant and clinically meaningful improvements in lung functions in treated patients compared to controls (Weiss et al., 2021). In a third example, the presence of *Aspergillus* in bronchoalveolar lavage samples from cystic fibrosis (CF) patients resulted in rapid MSC death, through actions of fungal gliotoxin on mitochondrial function (Abreu et al., 2020). As such, in the setting of a recent proof of concept safety trial of systemic bone marrow-derived MSC administration in CF patients, arguably patients with known Aspergillus lung infections, not uncommon in CF, should be excluded from subsequent efficacy investigations.

These observations and experience to date highlight both the promise and challenges of MSC-based cell therapies in lung regenerative medicine. This includes the still as yet unclear roles of endogenous lung MSCs in normal homeostasis and disease pathophysiology (Matthay et al., 2020; Bowdish et al., 2022; Kruk et al., 2021a; Kruk et al., 2021b). Further, MSCs are not the only cells being utilized in this context. Endothelial progenitor cells (EPCs) derived from both bone marrow as well as circulating sources have demonstrated efficacy in pre-clinical models of pulmonary hypertension and are currently under clinical investigation in pulmonary hypertension patients (Nagaya et al., 2003; Zhao et al., 2005; Foster et al., 2014; Suen et al., 2016). These are based on paracrine actions of the EPCs in part to stimulate angiogenesis and also include data on EPCs genetically engineered to produce angiogenic growth factors. The same considerations with respect to MSC therapeutics, source of origin, dose, dosing, etc, apply to EPCs and any other cell type being utilized for paracrine functional effects.

### Cell-based therapies: Structural repair

A landmark paper was published in 2002 in which bone marrow-derived cells appeared to structurally engraft as airway epithelial cells (Krause et al., 2001). This stimulated large-scale investigations of utilizing cell engraftment to repair damaged lung epithelium, notably in the context of cystic fibrosis. However, it was subsequently determined that technical and other errors provided misleading results and that engraftment of bone marrow-derived cells as structural lung cells was a rare occurrence of unlikely physiologic or clinical significance (Krause et al., 2001; Loi et al., 2006; Kotton et al., 2005)*.*

However, the concept of engraftment has been re-invigorated with increasing appreciation of the different populations of endogenous lung progenitor cells, both in the airway and alveolar epithelium, as well as advances in deriving lung epithelial cells from induced pluripotent-derived cells (iPSCs). Increasing data demonstrates that these cells can structurally and potentially functional engraft in pre-clinical models. One important caveat for these studies to date is that injury to the airway or alveolar epithelium is a predicate for engraftment. One simplistic explanation is that the damage provides “room” and allows the engrafting cells to integrate into previously intact cell monolayers. How this might translate into clinical practice is unclear at present but this is an exciting area of research.

### Lung support and assist devices: Functional repair

Regenerative medicine and engineering also incorporates developing devices that can augment or replace failing organs. This is well evidenced by examples of hemodialysis for chronic renal failure and left ventricular assist devices for heart failure. Such devices can also be utilized as bridges until organ transplantation or to improve quality of life in patients who do not qualify for organ transplants.

This is an area of necessary growth in respiratory medicine. Mechanical ventilators and other non-invasive ventilation approaches are powerful and well-established tools. However, for intractable respiratory failure, there are limited options. Extra-corporeal membrane oxygenation (ECMO) is an established tool for use in both neonatal and adult patients with intractable respiratory failure and has had increasing utilization in adult patients, particularly in the setting of the COVID-19 pandemic. However, ECMO is a complex approach requiring specialized centers and highly trained support personnel and is currently limited to relatively few tertiary care centers. Further ECMO can be complicated by clotting and bleeding in the circuitry as well as by infection. ECMO is also limited to short term use in intensive care settings; there are no current available device options for long term use in ambulatory patients on lung transplant waiting lists or who will not qualify for transplantation (Johnson et al., 2022).

There are several devices undergoing development and investigation by both academic and commercial entities (Syed et al., 2021). This includes a novel respiratory assist device based on decellularized bird lungs (Wrenn et al., 2018). However, there is much room for innovation and advancement.

### *Ex Vivo* generation of gas exchange tissues: Structural and functional repair

While lung transplantation is available and increasingly successful for patients with end-stage lung diseases, there remains a significant shortage of suitable donor organs. Lung transplantation is also complicated by still unacceptably high rates of both acute and chronic rejection. Accordingly, extensive effort has taken place over the past approximate 15 years to develop functional gas exchange tissue that can be implanted to replace defective lungs. This has led to development of a number of novel technical approaches that will be overviewed here.

### Lung de- and recellularization

Organ de- and recellularization has been widely investigated in a number of different tissues as a means to develop functional tissue *ex vivo* (Crapo et al., 2011). The overall goal is to utilize the native 3-dimensional (3D) extracellular matrix (ECM) as a scaffold for re-populating with organ-specific cells to develop functional organs and tissues. A variety of approaches are utilized to remove native cells and cellular debris including chemical, for example ionic and non-ionic detergents, and physical, for example multiple freeze thaws (reviewed in 51). With respect to lung, several seminal papers were published in 2010, based on an original report in 1986, that detailed detergent-based decellularization of rodent lungs and subsequent initial attempts at recellularization and implantation into rodent models (Lwebuga-Mukasa et al., 1986; Ott et al., 2010; Petersen et al., 2010; Price et al., 2010). Another report from that time investigated multiple freeze thaws as a model for decellularization (Cortiella et al., 2010). These proof-of concept studies provided a platform for a large number of subsequent studies through which significant progress has been made including ability to engraft multiple cell types and maintain viability for up to several weeks (Daly et al., 2012; Wallis et al., 2012; Bonenfant et al., 2013; Sokocevic et al., 2013; Wagner et al., 2014a; Wagner et al., 2014b).

However, significant challenges remain including the ability to fully repopulate and cover the entire ECM surface area and the question of having the proper cells in the proper location, i.e., alveolar in alveoli, airway in airways, etc. Significant parallel issues have been developing sources of cells to use in re-population strategies with increasing focus on utilizing lung endogenous progenitor cells and/or IPSC-derived cells as stem or progenitor populations with the goal of having these expand to large numbers and appropriately differentiate. One particular potential advantage of using lung progenitor or iPSC-derived cells is that they can be obtained from the eventual transplant recipient thus resulting in autologous tissue for use in transplantation. The working hypothesis here is that immune epitopes on the ECM that could potentially trigger rejection will either be removed during the decellularization process or covered by the seeded autologous cells. While an attractive hypothesis, direct studies addressing this have yet to be performed.

Efforts towards optimizing recellularization further depend in large part on environmental cues such as ECM composition and stiffness (Melo et al., 2014). Mechanical forces, i.e., cyclic mechanical stretch (“breathing”) for directing differentiation into airway or alveolar cells, or shear forces (vascular blood flow) for directing differentiation into pulmonary vascular endothelial cells, are increasingly being incorporated into re-population schemes. The development of novel bioreactor technology has also progressed enabling incorporation of mechanical forces but significant challenges remain in keeping the re-populating tissues alive for the weeks-months that may be required for full recellularization. Although the primary goal of functional *ex vivo* lung tissue is to provide gas exchange, immune regulatory actions of the lungs are also important functions. However, re-populating decellularized scaffolds with immune and inflammatory cells, such as macrophages, in addition to epithelial, vascular endothelial, and stromal cells, remains less well explored.

Thus, despite significant progress, a truly functional *ex vivo* organ capable of gas exchange as well as other immune regulating functions of the lung has not yet been developed. Further, even if this becomes technically achievable, there may still be not enough donor human lungs available to provide decellualrized scaffolds for use. Currently lungs are predominantly from those donor lungs that do not reach standards for use in transplantation or alternatively from autopsy. This has led to investigation of xenogeneic sources, primarily from pig lungs, as potential scaffolds for re-population with human lung cells (Platz et al., 2016; Gasek et al., 2021). As discussed previously, other postulated uses of other sources of lungs for decellularization include using decellularized bird lungs repopulated with human lung cells as artificial gas exchange (Wrenn et al., 2018). Further, even though there remains significant room for innovation and improvement in lung de- and recellularization approaches, decellularized organs have proven to be valuable experimental tools to study diseased ECM and cell-ECM interactions. For example, decellularized lungs from patients with COPD or IPF have provided significant insight into disease pathogenesis and the role of the ECM and disordered cell-matrix interactions in disease processes (Booth et al., 2012; Wagner et al., 2014b; Ahrman et al., 2018; Hoffman et al., 2023).

### Organoid and hydrogel approaches: Structural and functional repair

Three-dimensional cultures of cells in a supporting matrix has been an exploding field over the past few years. In contrast to standard two-dimensional (2D) tissue culture, 3D culturing proves a platform for 3D tissue formation and growth, in many cases resulting in miniature versions of the target organ or tissue (Saldin et al., 2017; Giobbe et al., 2019). These organoid culture approaches have provided a wealth of new information and studies including those for primary lung, lung progenitor cell, and iPSC-derived lung cells (Pouliot et al., 2016; De Hilster et al., 2020; Petrou et al., 2020; Pouliot et al., 2020; Nizamoglu et al., 2022; Marhuenda et al., 2022b; Saleh et al., 2022). These have also offered more sophisticated technologies with which to study interactions between different cell types co-seeded into the matrices as well as the influence of the 3D environmental niche as the 3D matrices can be produced with tunable stiffness and can also undergo cyclic mechanical strain (Pouliot et al., 2016; De Hilster et al., 2020; Petrou et al., 2020; Pouliot et al., 2020; Nizamoglu et al., 2022; Marhuenda et al., 2022b; Saleh et al., 2022).

Organoid culture approaches also offer a powerful opportunity to study the influence of the surrounding ECM composition on cell behaviors. However, to date, most organoid culture work, including those utilizing lung cells, have primarily utilized Matrigel™, a non-relevant ECM derived from cancerous mouse tissue typically utilized for stem and cancer cell proliferation, and thus, an insufficient ECM to replicate the *in situ* human lung environment (Petrou et al., 2020). However, recent studies have highlighted the potential role of physiologically relevant ECM on type 2 alveolar epithelial cells (AT2) differentiation (Alysandratos et al., 2022; Nizamoglu et al., 2022; Sucre et al., 2022). As such, several groups have recently been decellularized lungs for hydrogel formation and cell culture (Hughes et al., 2010; Pouliot et al., 2016; De Hilster et al., 2020; Petrou et al., 2020; Pouliot et al., 2020; Uhl et al., 2020; Alysandratos et al., 2022; Nizamoglu et al., 2022; Marhuenda et al., 2022b; Saleh et al., 2022; Sucre et al., 2022). Used in place of Matrigel™, the hydrogels are a unique culture model that provide a native and thus more relevant matrix. The lung-derived hydrogels can be produced not just from the entire lung but from airway, vascular, and alveolar-enriched regions obtained by dissection of decellularized lungs (Hoffman et al., 2023), this providing an opportunity to investigate regional differences in cell-matrix interactions. Further, hydrogels can be produced from decellularized diseased lungs providing additional novel model investigative systems (Petrou et al., 2020; Uhl et al., 2020; Saleh et al., 2022; Hoffman et al., 2023). These are powerful investigative tools that will provide novel information that in part can be utilized to improve recellularization schemes for decellularized lungs.

### 3D bioprinting: Structural repair

3D bioprinting has an increasingly important role in replacing damaged or diseased structural tissues with successful clinical applications in fields such as orthopedics and cosmetic repair. With respect to respiratory tissues, 3D-bioprinted tracheas and large airways have a growing potential clinical role (Ke et al., 2019; Mahfouzia et al., 2021; Park et al., 2021; Huo et al., 2022). However, there are a number of challenges including choice of matrix material to be used in the bioprinting process as well as effective vascularization and epithelialization of the bioprinted structure (Galliger et al., 2019; De Santis et al., 2021; Falcones et al., 2021). For use in congenital tracheal or airway defects, another consideration is whether the 3D-bioprinted structure will grow as the neonatal or infant recipient grows. While there is good progress to date, challenges in 3D bioprinting tissues that might participate in gas exchange result from several considerations mostly relating to the complex anatomic and physiologic lung structure-function relationship (Berg et al., 2021; Kang et al., 2021). Increasing sophistication of 3D bioprinting approaches, for example including use of techniques such as freeform reversible embedding of suspended hydrogels (FRESH) and incorporation of stereolithography along with advances in printer programming have allowed for ever more fine resolution of distal airways and potentially alveolar structures on a nano and micrometer scale (Sun et al., 2021). An exciting proof of concept study demonstrated that a 3D bioprinted vascular network on a micron scale, produced utilizing stereolithography and a light sensitive hydrogel that allowed for cell embedding could function in gas exchange (Grigoryan et al., 2019).

Other considerations include the bioink utilized in the printing process (Galliger et al., 2019; De Santis et al., 2021; Falcones et al., 2021). Will it mimic the native ECM composition, stiffness, and deformability (stretchability)? Of necessity, these will need to be varied during the bioprinting process in order to mimic the native lung structure. Similarly, the cells incorporated into the bioprinting process will need to be appropriately located in the relevant anatomic compartment and region. Alternative approaches include seeding cells into pre-printed scaffolds but the same challenges arise as for re-populating decellularized whole lung scaffolds. Can the distal bioprinted lung be effectively ventilated and undergo according cyclic mechanical strain? These are all substantial bioengineering challenges and there as yet no effective 3D-printed lungs. One alternative approach is to 3D-bioprint matrices that can be utilized in lung assist devices for gas exchange (Kang et al., 2021). These could be utilized for example in devices such as ECMO and obviate the need to fully mimic the lung anatomy.

### Other investigative tools to study structural and functional repair

A number of powerful tools have developed and are still in evolution. While not geared towards eventual direct *in vivo* use, these have provided platforms that underlie other attempts at structural and functional repair.

### Lung-on-a-Chip

Organ-on-a-chip model systems have developed for a number of organs and tissues. The first descriptions of lung-on-a-chip models highlights the general approach: cells are grown on either one or both sides of a biomimetic semi-permeable membrane in a small enclosed chamber. A general approach is to have epithelial cells on one side and vascular endothelial cells on the other (Huh et al., 2010; Huh et al., 2013). Microfluidic chambers on both compartments allow for gas and/or fluid flow and the biomimetic membranes can also undergo cyclic mechanical stretch (Zamprogno et al., 2021). Further innovative approaches have included incorporating lung stem and progenitor cells as well as primary lung epithelial and endothelial cells cultured on the membranes (Nawroth et al., 2019). These are powerful systems with which to study cell behaviors, cell-cell interactions, cell-matrix interactions, and the effects of environmental, such as tobacco smoke exposure, and physical forces on cell behaviors (Huh et al., 2012; Benam et al., 2016; Nawroth et al., 2020; Plebani et al., 2020; Bai et al., 2022). These, along with organoid model systems, are also powerful tools for evaluating drug effects and also for evaluating inflammatory and immune responses as both immune cells as well as infectious agents can be added to the culture systems (Huh et al., 2012; Benam et al., 2016; Nawroth et al., 2020; Plebani et al., 2020; Bai et al., 2022; Marhuenda et al., 2022a).

### Precision cut lung slices (PCLS)

Culturing thin sections of lung tissue has been an approach used for many years, primarily to assess actions of pharmacologic agents on airway smooth muscle contraction and relaxation [reviewed in (Alsafadi et al., 2020)]. However, one longstanding problem has been the viability of the cultures as cells, particularly those in more central and less well perfused regions of the cultures die within relatively short time periods. Another issue has been uniformity of the tissue slices. However, recent improvements in techniques for both producing and maintaining uniform slices have led to increased utility of this methodology for studying a range of biologic processes (Bailey et al., 2020; Stegmayr et al., 2021; Stegmayr and Wagner, 2021; Rosmark et al., 2022). As with lung-on-a-chip technologies, PCLS approaches offer strong models for studying cell behaviors, particularly in response to pharmacologic interventions or as short-term disease models. Continued improvements in technologies are necessary and desirable to further increase the utility of these approaches.

### Imaging and analytical techniques

Powerful advances in imaging and visualization techniques have provided further insights into processes involved in lung repair and regeneration [reviewed in (Ikonomou et al., 2020; Ikonomou et al., 2022)]. This includes both new techniques for cell and tissue labeling including multicolor multicomponent labeling of single cells that allows for detailed dynamic monitoring of cell behaviors *in situ* utilizing conventional fluoresence or confocal microscopy [reviewed in (Ikonomou et al., 2020; Ikonomou et al., 2022)]. In parallel a range of sophisticated imaging modalities have developed which allow for greater spatial and cellular resolution. One example of a relatively simple yet effective technique is termed inflation ie, infusion of fluorescently labelled antibodies in tissues of intact organs (Alsafadi et al., 2022). As indicated by the title, a continuous inflation-based infusion of antibodies provides opportunity to study 3-dimensional distribution of the cells or proteins of interest as compared to traditional immunostaining of mounted histologic sections.

Light sheet fluorescence microscopy is a technique that utilizes a plane of light to optically section and view tissues with subcellular resolution, essentially functioning as a non-destructive microtome [reviewed in (Santi, 2011)]. As tissues are exposed to only a thin plane of light, photobleaching and phototoxicity is minimized compared to other imaging modalities. Other techniques including live cell single particle imaging and multimodal imaging are also powerful tools (Ikonomou et al., 2020; Ikonomou et al., 2022).

In parallel with imaging, increasing advances in “omics” technologies, including *in situ* genomics and proteomics, along with advances in machine learning, single cell RNA sequencing and development of multi-omics atlases including gene and protein lung maps, are all tools that will help advance lung regenerative medicine and engineering (Pothen et al., 2016; Jorba et al., 2019; Marklein et al., 2019; Andreu et al., 2021).

## Summary

Techniques and approaches developed to study lung regenerative medicine and engineering offer promise to both better understand normal homeostasis and disease pathophysiologies as well as to devise new therapeutics. However, there remain multiple challenges and thus opportunities for up-and-coming lung biologists, biomedical engineers, and pulmonary and critical care physicians. The dictum that “today’s science fiction is tomorrow’s science” is particularly appropriate in this rapidly moving field.

## References

[B1] Abreu S. C. Enes S. R. Dearbron J. Goodwin M. Coffey A. Borg Z. D. (2019). Lung inflammatory environments differentially alter mesenchymal stromal cell behavior. Am. J. Physiol. Lung Cell Mol. Physiol. 317 (6), L823–L831. 10.1152/ajplung.00263.2019 31553626 PMC6962599

[B2] Abreu S. C. Hampton T. H. Hoffman E. Dearborn J. Ashare A. Singh Sidhu K. (2020). Differential effects of the cystic fibrosis lung inflammatory environment on mesenchymal stromal cells. Am. J. Physiology - Lung Cell. Mol. Physiology 319 (6), L908–L925. 10.1152/ajplung.00218.2020 PMC7792680 32901521

[B3] Abreu S. C. Lopes-Pacheco M. Weiss D. J. Rocco P. R. M. (2021). Mesenchymal stromal cell-derived extracellular vesicles in lung diseases: Current status and perspectives. Front. Cell & Dev. Biol. 9, 600711. 10.3389/fcell.2021.600711 33659247 PMC7917181

[B4] Ahrman E. Hallgren O. Malmstrom L. Hedstrom U. Malmstrom A. Bjermer L. (2018). Quantitative proteomic characterization of the lung extracellular matrix in chronic obstructive pulmonary disease and idiopathic pulmonary fibrosis. J. Proteomics 189, 23–33. 10.1016/j.jprot.2018.02.027 29501846

[B5] Allers C. Sierralta W. D. Neubauer S. Rivera F. Minguell J. J. Conget P. A. (2004). Dynamic of distribution of human bone marrow-derived mesenchymal stem cells after transplantation into adult unconditioned mice. Transplantation 78 (4), 503–508. 10.1097/01.tp.0000128334.93343.b3 15446307

[B6] Alsafadi H. N. Uhl F. E. Pineda R. H. Bailey K. E. Rojas M. Wagner D. E. (2020). Applications and approaches for three-dimensional precision-cut lung slices. Disease modeling and drug discovery. Am. J. Respir. Cell & Mol. Biol. 62 (6), 681–691. 10.1165/rcmb.2019-0276TR 31991090 PMC7401444

[B7] Alsafadi H. N. Tamargo I. Neves da Silva I. A. Rehnberb E. Wagner D. Stegmayr J. (2022). inFLATION: INfusion of fluorescent labelled antibodies in tissue of intact OrgaNs. 17th Annual Meeting of the European Society for Molecular Imaging, Mar 15 - 18, 2022, Greece

[B8] Alysandratos K.-D. Garcia C. Rivas A. Yao C. Pessina P. Villacorta-Martin C. (2022). Impact of cell culture on the transcriptomic programs of primary and iPSC-derived human alveolar type 2 cells. BioRxiv, 2022. 10.1101/2022.02.08.479591 PMC9870086 36454643

[B9] Andreu I. Falcones B. Hurst S. Chahare N. Quiroga X. Le Roux A. L. (2021). The force loading rate drives cell mechanosensing through both reinforcement and cytoskeletal softening. Nat. Commun. 12 (1), 4229. 10.1038/s41467-021-24383-3 34244477 PMC8270983

[B10] Armitage J. Tan D. B. A. Troedson R. Young P. Lam K. V. Shaw K. (2018). Mesenchymal stromal cell infusion modulates systemic immunological responses in stable COPD patients: a phase I pilot study. Eur. Respir. J. 51 (3), 1702369. 10.1183/13993003.02369-2017 29348155

[B11] Bai H. Si L. Jiang A. Belgur C. Zhai Y. Plebani R. (2022). Mechanical control of innate immune responses against viral infection revealed in a human lung alveolus chip. Nat. Commun. 13 (1), 1928. 10.1038/s41467-022-29562-4 35396513 PMC8993817

[B12] Bailey K. E. Pino C. Lennon M. L. Lyons A. Jacot J. G. Lammers S. R. (2020). Embedding of precision-cut lung slices in engineered hydrogel biomaterials supports extended *ex vivo* culture. Am. J. Respir. Cell & Mol. Biol. 62 (1), 14–22. 10.1165/rcmb.2019-0232MA 31513744 PMC6938134

[B13] Benam K. H. Novak R. Nawroth J. Hirano-Kobayashi M. Ferrante T. C. Choe Y. (2016). Matched-comparative modeling of normal and diseased human airway responses using a microengineered breathing lung chip. Cell Syst. 3 (5), 456–466. 10.1016/j.cels.2016.10.003 27894999

[B14] Berg J. Weber Z. Fechler-Bitteti M. Hocke A. C. Hippenstiel S. Elomaa L. (2021). Bioprinted multi-cell type lung model for the study of viral inhibitors. Viruses 13 (8), 1590. 10.3390/v13081590 34452455 PMC8402746

[B15] Bonenfant N. Sokocevic D. Wagner D. E. Borg Z. D. Lathrop M. Lam Y. W. (2013). The effects of storage and sterilization on de-cellularized and Re-cellularized whole lung. Biomaterials 34 (13), 3231–3245. 10.1016/j.biomaterials.2013.01.031 23380353 PMC4201372

[B16] Booth A. J. Hadley R. Cornett A. M. Dreffs A. A. Matthes S. A. Tsui J. L. (2012). Acellular normal and fibrotic human lung matrices as a culture system for *in vitro* investigation. Am. J. Respir. Crit. Care Med. 186 (9), 866–876. 10.1164/rccm.201204-0754OC 22936357 PMC3530219

[B17] Bowdish M. E. Barkauskas C. E. Overbey J. R. Gottlieb R. L. Osman K. Duggal A. (2022). A randomized trial of mesenchymal stromal cells for moderate to severe ARDS from COVID-19. Am. J. Respir. Crit. Care Med. 10.1164/rccm.202201-0157OC PMC9896641 36099435

[B18] Cortiella J. Niles J. Cantu A. Brettler A. Pham A. Vargas G. (2010). Influence of acellular natural lung matrix on murine embryonic stem cell differentiation and tissue formation. Tissue Eng. Part A 16 (8), 2565–2580. 10.1089/ten.tea.2009.0730 20408765

[B19] Crapo P. M. Gilbert T. W. Badylak S. F. (2011). An overview of tissue and whole organ decellularization processes. Biomaterials 32 (12), 3233–3243. 10.1016/j.biomaterials.2011.01.057 21296410 PMC3084613

[B20] Cruz F. F. Borg Z. D. Goodwin M. Sokocevic D. Wagner D. E. Coffey A. (2015). Systemic administration of human bone marrow-derived mesenchymal stromal cell extracellular vesicles ameliorates Aspergillus hyphal extract-induced allergic airway inflammation in immunocompetent mice. Stem Cells Transl. Med. 4 (11), 1302–1316. 10.5966/sctm.2014-0280 26378259 PMC4622402

[B21] Cruz F. F. Borg Z. D. Goodwin M. Sokocevic D. Wagner D. McKenna D. H. (2015). Freshly thawed and continuously cultured human bone marrow-derived mesenchymal stromal cells comparably ameliorate allergic airways inflammation in immunocompetent mice. Stem Cells Transl. Med. 4 (6), 615–624. 10.5966/sctm.2014-0268 25925837 PMC4449099

[B22] Daly A. B. Wallis J. M. Borg Z. D. Bonvillain R. W. Deng B. Ballif B. A. (2012). Initial binding and recellularization of decellularized mouse lung scaffolds with bone marrow-derived mesenchymal stromal cells. Bone Marrow-Derived Mesenchymal Stromal Cells Tissue Eng. Part A 18 (1-2), 1–16. 10.1089/ten.TEA.2011.0301 21756220 PMC4066256

[B23] De Hilster R. H. J. Sharma P. K. Jonker M. R. White E. S. Gercama E. A. Roobeek M. (2020). Human lung extracellular matrix hydrogels resemble the stiffness and viscoelasticity of native lung tissue. Am. J. Physiol. - Lung Cell. Mol. Physiol. 318, L698–L704. 10.1152/ajplung.00451.2019 32048864 PMC7191637

[B24] De Santis M. M. Alsafadi H. N. Tas S. Bolukbas D. A. Prithiviraj S. Da Silva I. A. N. (2021). Extracellular-matrix-reinforced bioinks for 3D bioprinting human tissue. Adv. Mater. 33 (3), e2005476. 10.1002/adma.202005476 33300242 PMC11469085

[B25] Dilogo I. H. Aditianingsih D. Sugiarto A. Burhan E. Damayanti T. Sitompul P. A. (2021). Umbilical cord mesenchymal stromal cells as critical COVID-19 adjuvant therapy: A randomized controlled trial. Stem Cells Transl. Med. 10 (9), 1279–1287. 10.1002/sctm.21-0046 34102020 PMC8242692

[B26] Enes S. R. Hampton T. H. Barua J. McKenna D. H. dos Santos C. C. Amiel E. (2021). Healthy versus inflamed lung environments differentially effect MSCs. Eur. Respir. J. 58 (4), 2004149. 10.1183/13993003.04149-2020 33795318 PMC8543758

[B27] Falcones B. Sanz H. Marhuenda E. Mendizabal I. Cabrera I. Malandain N. (2021). Bioprintable lung extracellular matrix hydrogel scaffolds for 3D culture of mesenchymal stromal cells. Polymers 13, 2350. 10.3390/polym13142350 34301107 PMC8309540

[B28] Foster W. S. Suen C. M. Stewart D. J. (2014). Regenerative cell and tissue-based therapies for pulmonary arterial hypertension. Can. J. Cardiol. 30 (11), 1350–1360. 10.1016/j.cjca.2014.08.022 25442435

[B29] Francois M. Copland I. B. Yuan S. Romieu-Mourez R. Waller E. K. Galipeau J. (2012). Cryopreserved mesenchymal stromal cells display impaired immunosuppressive properties as a result of heat-shock response and impaired interferon-gamma licensing. Cytotherapy 14 (2), 147–152. 10.3109/14653249.2011.623691 22029655 PMC3279133

[B30] Galipeau J. Sensébé L. (2018). Mesenchymal stromal cells: Clinical challenges and therapeutic opportunities. Cell Stem Cell 22 (6), 824–833. 10.1016/j.stem.2018.05.004 29859173 PMC6434696

[B31] Galipeau J. Krampera M. Leblanc K. Nolta J. A. Phinney D. G. Shi Y. (2021). Mesenchymal stromal cell variables influencing clinical potency: The impact of viability, fitness, route of administration and host predisposition. Cytotherapy 23 (5), 368–372. 10.1016/j.jcyt.2020.11.007 33714704 PMC11708105

[B32] Galleu A. Riffo-Vasquez Y. Trento C. Lomas C. Dolcetti L. Cheung T. S. (2017). Apoptosis in mesenchymal stromal cells induces *in vivo* recipient-mediated immunomodulation. Sci. Transl. Med. 9 (416), eaam7828. 10.1126/scitranslmed.aam7828 29141887

[B33] Galliger Z. Vogt C. D. Panoskaltsis-Mortari A. (2019). 3D bioprinting for lungs and hollow organs. Transl. Res. J. Laboratory Clin. Med. 211, 19–34. 10.1016/j.trsl.2019.05.001 PMC6702089 31150600

[B34] Gasek N. Dearborn J. Enes S. R. Pouliot R. Louie J. Phillips Z. (2021). Comparative immunogenicity of decellularized wild type and alpha 1,3 galactosyltransferase knockout pig lungs. Biomaterials 276, 121029. 10.1016/j.biomaterials.2021.121029 34311317

[B35] Giobbe G. G. Crowley C. Luni C. Campinoti S. Khedr M. Kretzschmar K. (2019). Extracellular matrix hydrogel derived from decellularized tissues enables endodermal organoid culture. Nat. Commun. 10, 5658. 10.1038/s41467-019-13605-4 31827102 PMC6906306

[B36] Grigoryan B. Paulsen S. J. Corbett D. C. Sazer D. W. Fortin C. L. Zaita A. J. (2019). Multivascular networks and functional intravascular topologies within biocompatible hydrogels. Science 364 (6439), 458–464. 10.1126/science.aav9750 31048486 PMC7769170

[B37] Hoffman E. T. Uhl F. E. Asarian L. Deng B. Becker C. Uriarte J. J. (2023). Regional and disease specific human lung extracellular matrix composition. Biomaterials 293, 121960. in press 2023. 10.1016/j.biomaterials.2022.121960 36580718 PMC9868084

[B38] Hughes C. S. Postovit L. M. Lajoie G. A. (2010). Matrigel: A complex protein mixture required for optimal growth of cell culture. Proteomics 10, 1886–1890. 10.1002/pmic.200900758 20162561

[B39] Huh D. Matthews B. D. Mammoto A. Montoya-Zavala M. Hsin H. Y. Ingber D. E. (2010). Reconstituting organ-level lung functions on a chip. Science 328 (5986), 1662–1668. 10.1126/science.1188302 20576885 PMC8335790

[B40] Huh D. Leslie D. C. Matthews B. D. Fraser J. P. Jurek S. Hamilton G. A. (2012). A human disease model of drug toxicity-induced pulmonary edema in a lung-on-a-chip microdevice. Sci. Transl. Med. 4 (159), 159ra147. 10.1126/scitranslmed.3004249 PMC8265389 23136042

[B41] Huh D. Kim H. J. Fraser J. P. Shea D. E. Khan M. Bahinski A. (2013). Microfabrication of human organs-on-chips. Nat. Protoc. 8 (11), 2135–2157. 10.1038/nprot.2013.137 24113786

[B42] Huo Y. Xu Y. Wu X. Gao E. Zhan A. Chen Y. (2022). Functional trachea reconstruction using 3D-bioprinted native-like tissue architecture based on designable tissue-specific bioinks. Adv. Sci. 9 (29), e2202181. 10.1002/advs.202202181 PMC9561786 35882628

[B43] Ikonomou L. Wagner D. E. Gilpin S. E. Weiss D. J. Ryan A. L. (2020). Technological advances in study of lung regenerative medicine: Perspective from the 2019 Vermont lung stem cell conference. Cytotherapy 22 (10), 519–520. 10.1016/j.jcyt.2020.04.001 32507605 PMC12908199

[B44] Ikonomou L. Magnusson M. Dries R. Herzog E. Hynds R. BorokPark Z. J-A. (2022). Stem cells, cell therapies, and bioengineering in lung biology and disease 2021. Am. J. Physiology - Lung Cell. Mol. Physiology 323 (3), L341–L354. 10.1152/ajplung.00113.2022 PMC9484991 35762622

[B45] Islam D. Huang Y. Fanelli V. Delsedime L. Wu S. Khang J. (2019). Identification and modulation of microenvironment is crucial for effective mesenchymal stromal cell therapy in acute lung injury. Am. J. Respir. Crit. Care Med. 199 (10), 1214–1224. 10.1164/rccm.201802-0356OC 30521764

[B46] Johnson B. Dobkin S. L. Josephson M. (2022). Extracorporeal membrane oxygenation as a bridge to transplant in neonates with fatal pulmonary conditions: A review. Paediatr. Respir. Rev. 44, 31–39. 10.1016/j.prrv.2022.11.001 36464576

[B47] Jorba I. Beltran G. Falcones B. Suki B. Farre R. Garcia-Aznar J. M. (2019). Nonlinear elasticity of the lung extracellular microenvironment is regulated by macroscale tissue strain. Acta Biomater. 92, 265–276. 10.1016/j.actbio.2019.05.023 31085362 PMC6701712

[B48] Kang D. Park J. A. Kim W. Kim S. Lee H. R. Kim W. J. (2021). All-inkjet-printed 3D alveolar barrier model with physiologically relevant microarchitecture. Adv. Sci. 8 (10), 2004990. 10.1002/advs.202004990 PMC8132150 34026463

[B49] Ke D. Yi H. Est-Witte S. George S. Kengla C. Lee S. J. (2019). Bioprinted trachea constructs with patient-matched design, mechanical and biological properties. Biofabrication 12 (1), 015022. 10.1088/1758-5090/ab5354 31671417

[B50] Khoury M. Cuenca J. Cruz F. F. Figueroa F. E. Rocco P. R. M. Weiss D. J. (2020). Current status of cell-based therapies for respiratory virus infections. Eur. Respir. J. 55 (6), 2000858. 10.1183/13993003.00858-2020 32265310 PMC7144273

[B51] Kirkham A. M. Bailey A. J. M. Monaghan M. Shorr R. Lalu M. M. Fergusson D. A. (2022). Updated living systematic review and meta-analysis of controlled trials of mesenchymal stromal cells to treat COVID-19: A framework for accelerated synthesis of trial evidence for rapid approval – FASTER approval. Stem Cells Transl. Med. 11, 675–687. in press. 10.1093/stcltm/szac038 35758400 PMC9299509

[B52] Kotton D. N. Fabian A. J. Mulligan R. C. (2005). Failure of bone marrow to reconstitute lung epithelium. Am. J. Respir. Cell & Mol. Biol. 33 (4), 328–334. 10.1165/rcmb.2005-0175RC 15961722 PMC2715341

[B53] Krampera M. Cosmi L. Angeli R. Pasini A. Liotta F. Andreini A. (2006). Role for interferon-gamma in the immunomodulatory activity of human bone marrow mesenchymal stem cells. Stem Cells 24 (2), 386–398. 10.1634/stemcells.2005-0008 16123384

[B54] Krause D. S. Theise N. D. Collector M. I. Henegariu O. Hwang S. Gardner R. (2001). Multi-organ, multi-lineage engraftment by a single bone marrow-derived stem cell. Cell 105 (3), 369–377. 10.1016/s0092-8674(01)00328-2 11348593

[B55] Kruk D. M. L. W. Wisman M. Bruin H. G. Lodewijk M. E. Hof D. J. Borghuis T. (2021a). Abnormalities in reparative function of lung-derived mesenchymal stromal cells in emphysema. Am. J. Physiology - Lung Cell. Mol. Physiology 320 (5), L832–L844. 10.1152/ajplung.00147.2020 33656381

[B56] Kruk D. M. L. W. Wisman M. Noordhoek J. A. Nizamoglu M. Jonker M. R. de Bruin H. G. (2021b). Paracrine regulation of alveolar epithelial damage and repair responses by human lung-resident mesenchymal stromal cells. Cells 10 (11), 2860. 10.3390/cells10112860 34831082 PMC8616441

[B57] Kusuma G. D. Carthew J. Lim R. Frith J. E. (2017). Effect of the microenvironment on mesenchymal stem cell paracrine signaling: Opportunities to engineer the therapeutic effect. Stem Cells Dev. 26 (9), 617–631. 10.1089/scd.2016.0349 28186467

[B58] Lanzoni G. Linetsky E. Correa D. Messinger Cayetano S. Alvarez R. A. Kouroupis D. (2021). Umbilical cord mesenchymal stem cells for COVID-19 acute respiratory distress syndrome: A double-blind, phase 1/2a, randomized controlled trial. Stem Cells Transl. Med. 10, 660–673. 10.1002/sctm.20-0472 33400390 PMC8046040

[B59] Loi R. Beckett T. Goncz K. K. Suratt B. T. Weiss D. J. (2006). Limited restoration of cystic fibrosis lung epithelium *in vivo* with adult bone marrow-derived cells. Am. J. Resp. Crit. Care Med. 173, 171–179. 10.1164/rccm.200502-309OC 16179642 PMC2662986

[B60] Lu K. Geng S. T. Tang S. Yang H. Xiong W. Xu F. (2022). Clinical efficacy and mechanism of mesenchymal stromal cells in treatment of COVID-19. Stem Cell Res. Ther. 13, 61–15. 10.1186/s13287-022-02743-0 35130977 PMC8822653

[B61] Lwebuga-Mukasa J. S. Ingbar D. H. Madri J. A. (1986). Repopulation of a human alveolar matrix by adult rat type II pneumocytes *in vitro*. A novel system for type II pneumocyte culture. Exptl Lung Res. 162 (2), 423–435. 10.1016/0014-4827(86)90347-2 3510880

[B62] Mahfouzia S. H. Tali S. H. S. Amoabediny G. (2021). 3D bioprinting for lung and tracheal tissue engineering: Criteria, advances, challenges, and future directions. Bioprinting 21, e00124. 10.1016/j.bprint.2020.e00124

[B63] Mahida R. Y. Matsumoto S. Matthay M. A. (2020). Extracellular vesicles: A new frontier for research in acute respiratory distress syndrome. Am. J. Respir. Cell & Mol. Biol. 63 (1), 15–24. 10.1165/rcmb.2019-0447TR 32109144 PMC7328246

[B64] Marhuenda E. Villarino A. Narciso M. Elowsson L. Almendros I. Westergren-Thorsson G. (2022a). Development of a physiomimetic model of acute respiratory distress syndrome by using ECM hydrogels and organ-on-a-chip devices. Front. Pharmacol. 13, 945134. 10.3389/fphar.2022.945134 36188621 PMC9517737

[B65] Marhuenda E. Villarino A. Narciso M. L. Camprubi-Rimblas M. Farre R. Gavara N. (2022b). Lung extracellular matrix hydrogels enhance preservation of type II phenotype in primary alveolar epithelial cells. Int. J. Mol. Sci. 23 (9), 4888. 10.3390/ijms23094888 35563279 PMC9100165

[B66] Marklein R. A. Klinker M. W. Drake K. A. Polikowsky H. G. Lessey-Morillon E. C. Bauer S. R. (2019). Morphological profiling using machine learning reveals emergent subpopulations of interferon-gamma-stimulated mesenchymal stromal cells that predict immunosuppression. Cytotherapy 21 (1), 17–31. 10.1016/j.jcyt.2018.10.008 30503100

[B67] Matthay M. A. Zemans R. L. Zimmerman G. A. Arabi Y. M. Beitler J. R. Mercat A. (2019). Acute respiratory distress syndrome. Nat. Rev. Dis. Prim. 5 (1), 18. 10.1038/s41572-019-0069-0 30872586 PMC6709677

[B68] Matthay M. A. Calfee C. S. Zhuo H. Thompson B. T. Wilson J. G. Levitt J. E. (2019). Treatment with allogeneic mesenchymal stromal cells for moderate to severe acute respiratory distress syndrome (START study): A randomised phase 2a safety trial. Lancet Respir. Med. 7 (2), 154–162. 10.1016/S2213-2600(18)30418-1 30455077 PMC7597675

[B69] Matthay M. A. Arabi Y. M. Siegel E. R. Ware L. B. Bos L. D. J. Sinha P. (2020). Phenotypes and personalized medicine in the acute respiratory distress syndrome. Intensive Care Med. 46 (12), 2136–2152. 10.1007/s00134-020-06296-9 33206201 PMC7673253

[B70] Melo E. Garreta E. Luque T. Cortiella J. Nichols J. Navajas D. (2014). Effects of the decellularization method on the local stiffness of acellular lungs. Tissue Eng. - Part C. Methods 20 (5), 412–422. 10.1089/ten.TEC.2013.0325 24083889

[B71] Monsel A. Hauw-Berlemont C. Mebarki M. Heming N. Mayaux J. Nguekap Tchoumba O. (2022). Treatment of COVID-19-associated ARDS with mesenchymal stromal cells: A multicenter randomized double-blind trial. Crit. Care 26, 48. 10.1186/s13054-022-03930-4 35189925 PMC8860258

[B72] Nagaya N. Kangawa K. Kanda M. Uematsu M. Horio T. Fukuyama N. (2003). Hybrid cell-gene therapy for pulmonary hypertension based on phagocytosing action of endothelial progenitor cells. Circulation 108, 889–895. 10.1161/01.CIR.0000079161.56080.22 12835224

[B73] Nawroth J. C. Barrile R. Conegliano D. van Riet S. Hiemstra P. S. Villenave R. (2019). Stem cell-based Lung-on-Chips: The best of both worlds? Adv. Drug Deliv. Rev. 140, 12–32. 10.1016/j.addr.2018.07.005 30009883 PMC7172977

[B74] Nawroth J. C. Lucchesi C. Cheng D. Shukla A. Ngyuen J. Shroff T. (2020). A microengineered airway lung chip models key features of viral-induced exacerbation of asthma. Am. J. Respir. Cell & Mol. Biol. 63 (5), 591–600. 10.1165/rcmb.2020-0010MA 32706623

[B75] Nizamoglu M. de Hilster R. H. J. Zhao F. Sharma P. K. Borghuis T. Harmsen M. C. (2022). An *in vitro* model of fibrosis using crosslinked native extracellular matrix-derived hydrogels to modulate biomechanics without changing composition. Acta Biomater. 147, 50–62. 10.1016/j.actbio.2022.05.031 35605955

[B76] Ott H. C. Clippinger B. Conrad C. Schuetz C. Pomerantseva I. Ikonomou L. (2010). Regeneration and orthotopic transplantation of a bioartificial lung. Nat. Med. 16, 927–933. 10.1038/nm.2193 20628374

[B77] Park J. H. Ahn M. Park S. H. Kim H. Bae M. Park W. (2021). 3D bioprinting of a trachea-mimetic cellular construct of a clinically relevant size. Biomaterials 279, 121246. 10.1016/j.biomaterials.2021.121246 34775331 PMC8663475

[B78] Petersen T. H. Calle E. A. Zhao L. Lee E. J. Gui L. Raredon M. B. (2010). Tissue-engineered lungs for *in vivo* implantation. Science 329, 538–541. 10.1126/science.1189345 20576850 PMC3640463

[B79] Petrou C. L. D'Ovidio T. J. Bolukbas D. A. Tas S. Brown R. D. Allawzi A. (2020). Clickable decellularized extracellular matrix as a new tool for building hybrid-hydrogels to model chronic fibrotic diseases *in vitro* . J. Mater. Chem. B 8 (31), 6814–6826. 10.1039/d0tb00613k 32343292 PMC9020195

[B80] Platz J. Bonenfant N. R. Uhl F. E. Coffey A. L. McKnight T. Parsons C. (2016). Comparative decellularization and recellularization of wild-type and alpha 1,3 galactosyltransferase knockout pig lungs: A model for *ex vivo* xenogeneic lung bioengineering and transplantation. Tissue Eng. Part C Methods 22 (8), 725–739. 10.1089/ten.TEC.2016.0109 27310581 PMC4991572

[B81] Plebani R. Potla R. Soong M. Bai H. Izadifar Z. Jiang A. (2020). Modeling pulmonary cystic fibrosis in a human lung airway-on-a-chip. J. Cyst. Fibros. 21 (4), 606–615. 10.1016/j.jcf.2021.10.004 34799298

[B82] Polchert D. Sobinsky J. Douglas G. Kidd M. Moadsiri A. Reina E. (2008). IFN-gamma activation of mesenchymal stem cells for treatment and prevention of graft versus host disease. Eur. J. Immunol. 38 (6), 1745–1755. 10.1002/eji.200738129 18493986 PMC3021120

[B83] Pothen J. J. Rajendran V. Wagner D. Weiss D. J. Smith B. J. Ma B. (2016). A computational model of cellular engraftment on lung scaffolds. Biores Open Access 5 (1), 308–319. 10.1089/biores.2016.0031 27843709 PMC5107660

[B84] Pouliot R. A. Link P. A. Mikhaiel N. S. Schneck M. B. Valentine M. S. Kamga Gninzeko F. J. (2016). Development and characterization of a naturally derived lung extracellular matrix hydrogel. J. Biomed. Mat. Res. - Part A. 104, 1922–1935. 10.1002/jbm.a.35726 PMC7962169 27012815

[B85] Pouliot R. A. Young B. M. Link P. A. Park H. E. Kahn A. R. Shankar K. (2020). Porcine lung-derived extracellular matrix hydrogel properties are dependent on pepsin digestion time. Tissue Eng. - Part C. Methods 26 (6), 332–346. 10.1089/ten.TEC.2020.0042 32390520 PMC7310225

[B86] Price A. P. England K. A. Matson A. M. Blazar B. R. Panoskaltsis-Mortari A. (2010). Development of a decellularized lung bioreactor system for bioengineering the lung: The matrix reloaded. Tissue Eng. Part A 16, 2581–2591. 10.1089/ten.TEA.2009.0659 20297903 PMC2947435

[B87] Qu W. Wang Z. Hare J. M. Bu G. Mallea J. M. Pascual J. M. (2020). Cell‐based therapy to reduce mortality from COVID‐19: Systematic review and meta‐analysis of human studies on acute respiratory distress syndrome. Stem Cells Transl. Med. 9, 1007–1022. 10.1002/sctm.20-0146 32472653 PMC7300743

[B88] Romieu-Mourez R. Francois M. Boivin M. N. Bouchentouf M. Spaner D. E. Galipeau J. (2009). Cytokine modulation of TLR expression and activation in mesenchymal stromal cells leads to a proinflammatory phenotype. J. Immunol. 182 (12), 7963–7973. 10.4049/jimmunol.0803864 19494321

[B89] Rosmark O. Ibanez-Fonseca A. Thorsson J. Dellgren G. Hallgren O. Larsson Callerfelt A. K. (2022). A tunable physiomimetic stretch system evaluated with precision cut lung slices and recellularized human lung scaffolds. Front. Bioeng. Biotechnol. 10, 995460. 10.3389/fbioe.2022.995460 36263353 PMC9574011

[B90] Saldin L. T. Cramer M. C. Velankar S. S. White L. J. Badylak S. F. (2017). Extracellular matrix hydrogels from decellularized tissues: Structure and function. Acta Biomater. 49, 1–15. 10.1016/j.actbio.2016.11.068 27915024 PMC5253110

[B91] Saleh K. S. Hewawasam R. Serbedzija P. Blomberg R. Noreldeen S. E. Edelman B. (2022). Engineering hybrid-hydrogels comprised of healthy or diseased decellularized extracellular matrix to study pulmonary fibrosis. Cell. Mol. Bioeng. 15 (5), 505–519. 10.1007/s12195-022-00726-y 36444345 PMC9700547

[B92] Santi P. A. (2011). Light sheet fluorescence microscopy: A review. J. Histochem Cytochem 59 (2), 129–138. 10.1369/0022155410394857 21339178 PMC3201139

[B93] Sokocevic D. Bonenfant N. Wagner D. E. Borg Z. D. Lathrop M. Lam Y. W. (2013). The effect of age and emphysematous and fibrotic injury on the Re-cellularization of de-cellularized lungs. Biomaterials 34 (13), 3256–3269. 10.1016/j.biomaterials.2013.01.028 23384794 PMC4215729

[B94] Stegmayr J. Wagner D. E. (2021). The dawn of the omics era in human precision-cut lung slices. Eur. Respir. J. 58 (1), 2100203. 10.1183/13993003.00203-2021 34215663

[B95] Stegmayr J. Alsafadi H. N. Langwinski W. Niroomand A. Lindstedt S. Leigh N. D. (2021). Isolation of high-yield and -quality RNA from human precision-cut lung slices for RNA-sequencing and computational integration with larger patient cohorts. Am. J. Physiology - Lung Cell. Mol. Physiology 320 (2), L232–L240. 10.1152/ajplung.00401.2020 33112185

[B96] Sucre J. M. S. Bock F. Negretti N. M. Benjamin J. T. Gulleman P. M. Dong X. (2022). β1 integrin regulates alveolar epithelial cell differentiation following injury, BioRxiv.

[B97] Suen C. M. Zhai A. Lalu M. M. Welsh C. Levac B. M. Fergusson D. (2016). Efficacy and safety of regenerative cell therapy for pulmonary arterial hypertension in animal models: A preclinical systematic review protocol. Syst. Rev. 5, 89. 10.1186/s13643-016-0265-x 27225668 PMC4880876

[B98] Sun W. Schaffer S. Dai K. Yao L. Feinberg A. Webster-Wood V. (2021). 3D printing hydrogel-based soft and biohybrid actuators: A mini-review on fabrication techniques, applications, and challenges. Front. Robotics AI 8, 673533. 10.3389/frobt.2021.673533 PMC8117231 33996931

[B99] Syed A. Kerdi S. Qamar A. (2021). Bioengineering progress in lung assist devices. Bioengineering Basel 8, 89. 10.3390/bioengineering8070089 34203316 PMC8301204

[B100] Ting A. E. Baker E. K. Champagne J. Desai T. J. dos Santos C. C. Heijink I. H. (2022). Proceedings of the ISCT scientific signature series symposium, "Advances in cell and gene therapies for lung diseases and critical illnesses": International Society for Cell & Gene Therapy, Burlington VT, US, July 16, 2021. Cytotherapy 24 (8), 774–788. 10.1016/j.jcyt.2021.11.007 35613962

[B101] Uhl F. E. Zhang F. Pouliot R. A. Uriarte J. J. Rolandsson Enes S. Han X. (2020). Functional role of glycosaminoglycans in decellularized lung extracellular matrix. Acta Biomater. 102, 231–246. 10.1016/j.actbio.2019.11.029 31751810 PMC8713186

[B102] Wagner D. E. Bonenfant N. R. Sokocevic D. DeSarno M. Borg Z. Parsons C. (2014). Three-dimensional scaffolds of acellular human and porcine lungs for high throughput studies of lung disease and regeneration. Biomaterials 35 (9), 2664–2679. 10.1016/j.biomaterials.2013.11.078 24411675 PMC4215726

[B103] Wagner D. E. Bonenfant N. R. Parsons C. Sokocevic D. Borg Z. S. Lathrop M. (2014). Comparative decellularization and recellularization of normal versus emphysematous human lungs. Biomaterials 35 (10), 3281–3297. 10.1016/j.biomaterials.2013.12.103 24461327 PMC4215725

[B104] Wallis J. M. Borg Z. D. Daly A. B. Deng B. Ballif B. A. Allen G. B. (2012). Comparative assessment of detergent-based protocols for mouse lung de-cellularization and Re-cellularization. Tissue Eng. Part C 18 (6), 420–432. 10.1089/ten.tec.2011.0567 PMC3358122 22165818

[B105] Walter J. Ware L. B. Matthay M. A. (2014). Mesenchymal stem cells: Mechanisms of potential therapeutic benefit in ARDS and sepsis. Lancet Respir. Med. 2 (12), 1016–1026. 10.1016/S2213-2600(14)70217-6 25465643

[B106] Waterman R. S. Tomchuck S. L. Henkle S. L. Betancourt A. M. (2010). A new mesenchymal stem cell (MSC) paradigm: Polarization into a pro-inflammatory MSC1 or an immunosuppressive MSC2 phenotype. PLoS One 5 (4), e10088. 10.1371/journal.pone.0010088 20436665 PMC2859930

[B107] Weiss D. J. Casaburi R. Flannery R. LeRoux-Williams M. Tashkin D. P. (2013). A placebo-controlled, randomized trial of mesenchymal stem cells in COPD. Chest 143 (6), 1590–1598. 10.1378/chest.12-2094 23172272 PMC4694112

[B108] Weiss D. J. English K. Krasnodembskaya A. Isaza-Correa J. M. Hawthorne I. J. Mahon B. P. (2019). The necrobiology of mesenchymal stromal cells affects therapeutic efficacy. Front. Immunol. 10, 1228. 10.3389/fimmu.2019.01228 31214185 PMC6557974

[B109] Weiss D. J. Segal K. Casaburi R. Hayes J. Tashkin D. (2021). Effect of mesenchymal stromal cell infusions on lung function in COPD patients with high CRP levels. Respir. Res. 22 (1), 142. 10.1186/s12931-021-01734-8 33964910 PMC8106850

[B110] Wrenn S. M. Griswold E. D. Uhl F. E. Uriarte J. J. Park H. E. Coffey A. L. (2018). Avian lungs: A novel scaffold for lung bioengineering. PLoS One 13 (6), e0198956. 10.1371/journal.pone.0198956 29949597 PMC6021073

[B111] Zamprogno P. Wuthrich S. Achenbach S. Thoma G. Stucki J. D. Hobi N. (2021). Second-generation lung-on-a-chip with an array of stretchable alveoli made with a biological membrane. Commun. Biol. 4 (1), 168. 10.1038/s42003-021-01695-0 33547387 PMC7864995

[B112] Zhao Y. D. Courtman D. W. Deng Y. Kugathasan L. Zhang Q. Stewart D. J. (2005). Rescue of monocrotaline-induced pulmonary arterial hypertension using bone marrow-derived endothelial-like progenitor cells: Efficacy of combined cell and eNOS gene therapy in established disease. Circ. Res. 96, 442–450. 10.1161/01.RES.0000157672.70560.7b 15692087

[B113] Zhu Y. G. Feng X. M. Abbott J. Fang X. H. Hao Q. Monsel A. (2014). Human mesenchymal stem cell microvesicles for treatment of *Escherichia coli* endotoxin-induced acute lung injury in mice. Stem Cells 32 (1), 116–125. 10.1002/stem.1504 23939814 PMC3947321

[B114] Zhu R. Yan T. Feng Y. Liu Y. Cao H. Peng G. (2021). Mesenchymal stem cell treatment improves outcome of COVID-19 patients via multiple immunomodulatory mechanisms. Cell Res. 31 (12), 1244–1262. 10.1038/s41422-021-00573-y 34702946 PMC8546390

